# Compact Cas12 Systems for Multiplex Engineering of Plant Abiotic‐Stress Networks

**DOI:** 10.1002/ggn2.202500067

**Published:** 2026-06-15

**Authors:** Samar G. Thabet, Khairiah Mubarak Alwutayd, Amr Elkelish, Fatmah Ahmed Safhi, Mona F.A. Dawood, Ahmad M. Alqudah

**Affiliations:** ^1^ Department of Botany Faculty of Science Fayoum University Fayoum Egypt; ^2^ Department of Biology College of Science Princess Nourah Bint Abdulrahman University Riyadh Saudi Arabia; ^3^ Department of Biology College of Science Imam Mohammad Ibn Saud Islamic University (IMSIU) Riyadh Kingdom of Saudi Arabia; ^4^ Botany and Microbiology Department Faculty of Science Assiut University Assiut Egypt; ^5^ Biological Science Program, Department of Biological and Environmental Sciences College of Arts and Sciences Qatar University Doha Qatar

**Keywords:** abiotic‐stress networks, compact Cas12, miniature CRISPR nucleases, multiplex editing, plant genome editing, regulatory‐region engineering, viral vector delivery

## Abstract

Compact CRISPR‐Cas12 platforms, particularly miniature and hypercompact effectors such as Cas12f, Cas12j/CasPhi, and Cas12lambda, are emerging as delivery‐efficient tools for plant genome engineering because their reduced coding size facilitates viral‐replicon and other size‐constrained delivery routes while supporting dense guide multiplexing. Here, we argue that their highest value for crop improvement lies not simply in additional nuclease choices, but in programmable, systems‐level tuning of abiotic‐stress defense layers, including ion homeostasis, redox buffering, osmotic adjustment, abscisic acid (ABA)‐centered hormone signaling, and transcriptional control. We distinguish evidence already demonstrated in stable plants, transient plant assays, or protoplasts from approaches extrapolated from Cas9/Cas12a or non‐plant systems, and emphasize that many compact Cas12 applications remain platform‐building rather than field‐ready technologies. Current plant evidence supports Cas12a as a mature multiplex‐editing and transcriptional‐repression platform, Cas12f as a rapidly improving mini‐editor with stable and viral‐delivery demonstrations, and Cas12j/CasPhi as a hypercompact system with recent crop‐editing and base‐editing potential. By contrast, Cas12g, Cas12h, Cas12lambda, and TnpB‐like nucleases are best viewed as promising future modules until plant‐active implementations are broadly validated. We offer a forward‐looking roadmap in which compact Cas12 systems complement Cas9 and Cas12a by enabling rapid target triage, promoter and untranslated‐region engineering, transient CRISPR activation/interference, and stepwise assembly of durable sentinel edits. Translation will depend on improving efficiency and fidelity across genotypes, reducing mosaicism, documenting off‐target and structural variation in multiplex stacks, and aligning delivery strategies with regulatory expectations for transgene‐free crops.

## Graphical abstract

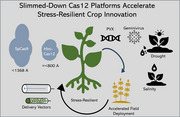

## 1 Introduction

Climate change is intensifying drought, salinity, temperature extremes, and nutrient/heavy‐metal toxicities, with major consequences for crop productivity. Soil salinization and waterlogging already affect more than 20% of the global irrigated area and may expand substantially without improved management [1], while drought remains a leading driver of yield instability and food insecurity [2]. The limiting factor for climate‐resilient crop design is therefore no longer only the discovery of stress‐responsive genes, but the ability to deploy fast, precise, and multiplexable interventions that reprogram stress‐response networks directly in elite germplasm. Genome editing, particularly CRISPR‐based systems, provides this capability by enabling targeted rewiring of native pathways rather than slow reshuffling of alleles through prolonged introgression [3]. Abiotic‐stress tolerance is intrinsically networked: individual stresses frequently impose osmotic, ionic, and oxidative components simultaneously, requiring coordinated responses across cells, tissues, and developmental stages. Key modules include ion homeostasis, which limits cytosolic Na+ toxicity and maintains K+ balance under salinity through transporter systems such as SOS1, HKT1 and NHX [4]; reactive oxygen species (ROS) detoxification, where antioxidant enzymes and protective proteins constrain oxidative injury while preserving redox signaling [5]; osmotic adjustment, supported by compatible solutes and osmotic‐stress pathways involving stress‐associated proteins (SAPs), aquaporins and osmolyte biosynthesis [6, 7]; abscisic acid (ABA) signaling, which integrates stomatal control, gene expression and osmolyte accumulation and can be tuned through PYL and PP2C components to improve water‐use efficiency [8]; and transcriptional reprogramming, where transcription factor (TF) families such as DREB, NAC and bZIP orchestrate downstream defences [9, 10]. Because these modules interact across scales, stress tolerance is typically polygenic and context‐dependent, which explains why single‐locus breeding gains are often modest [2]. CRISPR/Cas editing compresses this timeline by enabling direct modification of native circuits in elite cultivars. For example, promoter replacement at ARGOS8 in maize improved grain yield under drought by altering stress responsiveness [11], and CRISPR/Cas9 knockouts of negative regulators, including OsDST in rice and SlARF4 in tomato, enhanced drought avoidance or salinity tolerance without clear growth penalties [10, 12]. Durable climate resilience will therefore most often require multiplex designs that stack complementary modules, such as ion balance, ROS buffering, and water‐status control, rather than expecting large effects from a single edit. Cas12a (Cpf1) already contributes to this logic through a distinct TTTV protospacer‐adjacent motif (PAM), a crRNA‐only guide architecture, staggered cuts, and intrinsic processing of crRNA arrays, all of which expand editable space and simplify multiplexing [14, 15, 16]. Beyond knockouts, Cas12a has been adapted for transcriptional repression in plants, whereas plant CRISPR‐Act systems demonstrate the feasibility of multiplex gene activation; together, these platforms support programmable network tuning rather than single‐gene disruption [15, 17]. Miniature and hypercompact Cas12 variants, especially Cas12f and Cas12j/CasPhi, further reduce delivery constraints for plant viral vectors, geminiviral replicons, and compact T‐DNA constructs [18, 19, 20, 21, 22]. Thus, compact Cas12 systems should be framed as complements to Cas9 and Cas12a: they shift the engineering bottleneck from identifying stress genes to testing which combinations can be delivered, tuned, and fixed efficiently in elite backgrounds. Their smaller size supports rapid delivery, their guide‐array compatibility matches the polygenic nature of stress tolerance, and their targeting flexibility increases access to promoters, enhancers, and untranslated regions where agronomic gains can be achieved with reduced pleiotropic cost [23, 24].

## 2 Classification of the CRISPR‐Cas System

**FIGURE 1:**
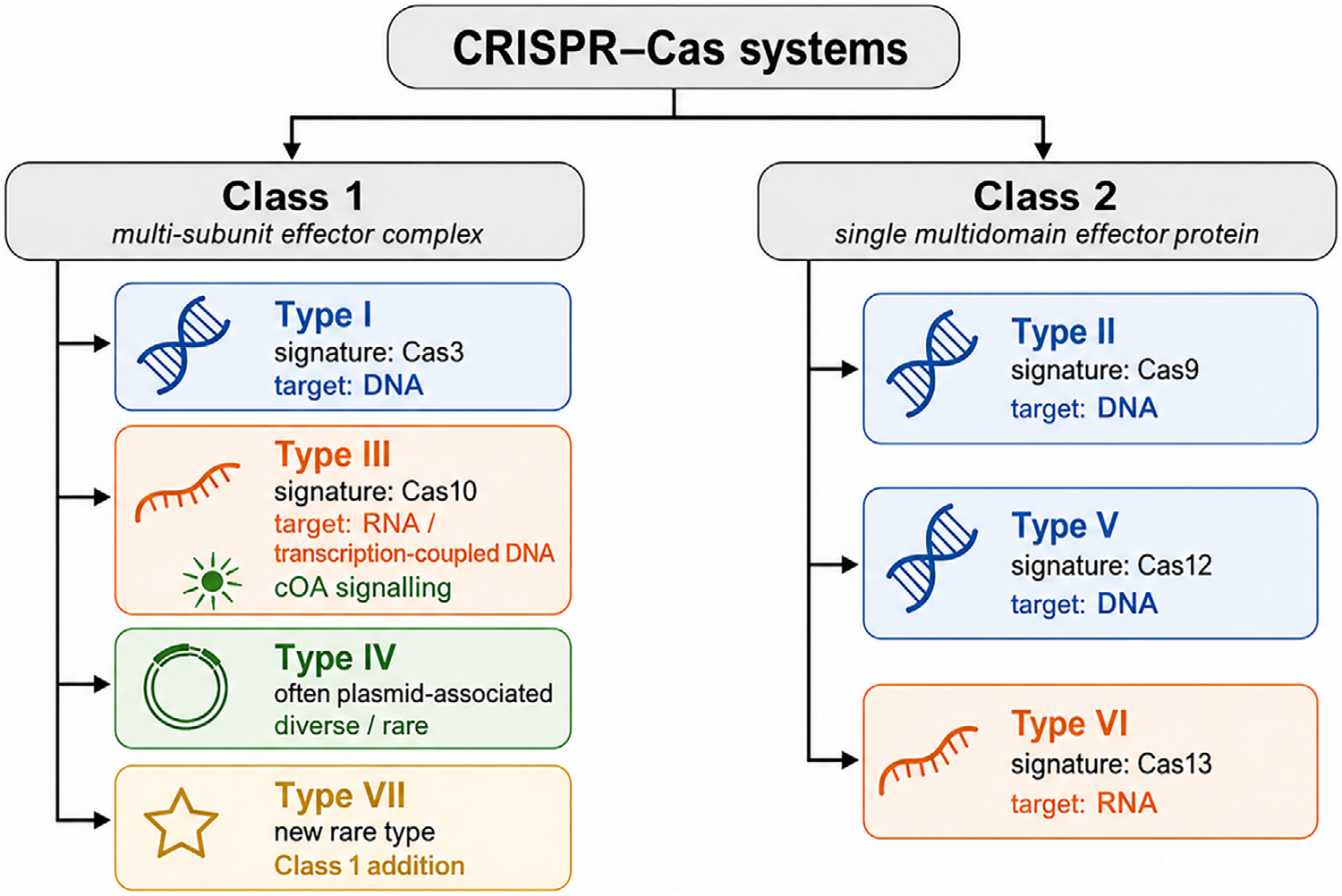
Classification of CRISPR–Cas systems.CRISPR–Cas systems are organized into two major architectural classes under the updated two‐class/seven‐type evolutionary framework. Class 1 systems use multi‐subunit effector complexes and include Type I, Type III, Type IV, and the recently recognized Type VII systems. Class 2 systems use single multidomain effector proteins and include Type II, Type V, and Type VI systems. The representative signature effectors and target preferences are shown for each type, including Cas3 for Type I DNA targeting, Cas10‐associated RNA/transcription‐coupled DNA targeting and cyclic oligoadenylate (cOA) signaling in Type III, Cas9 in Type II, Cas12 in Type V and Cas13 in Type VI. Type IV systems are frequently plasmid‐associated and diverse, whereas Type VII represents a rare Class 1 addition [28]. This image was created with BioRender (https://biorender.com/).

**FIGURE 2:**
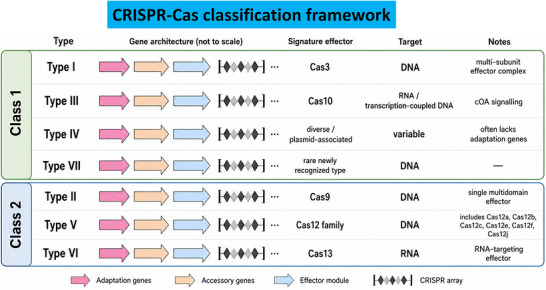
CRISPR–Cas classification framework. Schematic of the updated two‐class/seven‐type CRISPR–Cas classification framework, showing representative gene architecture, signature effectors, target molecules and key functional notes. Class 1 systems use multi‐subunit effector complexes and include Type I, Type III, Type IV, and Type VII systems, represented by Cas3‐mediated DNA targeting, Cas10‐associated RNA/transcription‐coupled DNA targeting and cOA signaling, plasmid‐associated diverse Type IV systems, and the rare newly recognized Type VII group. Class 2 systems use single multidomain effector proteins and include Type II, Type V, and Type VI systems, represented by Cas9, the Cas12 family, and Cas13, respectively. This image was created with BioRender (https://biorender.com/).

CRISPR‐Cas taxonomy is useful here only insofar as it explains why compact Cas12 effectors expand plant engineering options (Figure 1). Figure 1 introduces the updated two‐class/seven‐type evolutionary framework, in which Class 1 systems use multi‐subunit effector complexes and Class 2 systems use single multidomain effectors [27]. This distinction matters because most plant genome‐editing platforms rely on the compact expression of a single programmable nuclease. Figure 2 then translates the classification into a practical readiness map for plant biotechnology. Cas9 and Cas12a remain the mature reference systems; Cas12a is especially important because its crRNA‐only guide architecture, staggered DNA cleavage, and intrinsic crRNA‐array processing already support multiplex editing in plants and transcriptional repression [14, 15, 16, 31]. The miniature Type V branch is then separated by evidence level: Cas12f and engineered Cas12j/CasPhi have the strongest emerging plant evidence, whereas Cas12g, Cas12h, Cas12lambda, and TnpB‐like relatives remain promising but less mature for crop deployment [18, 19, 20, 21, 22, 33, 35, 36, 37, 38, 39, 40, 41, 42, 43, 44, 45, 46, 47, 48, 49, 52]. The most recent evolutionary classification recognizes two architectural classes, seven types, and 46 subtypes, reflecting continuing discovery of rare and derived variants [27]. Class 1 systems use multi‐protein surveillance complexes, whereas Class 2 systems condense guide binding, target recognition, and cleavage into single effector proteins. The latter architecture is the foundation of most plant genome‐editing platforms because a single coding sequence can be expressed with programmable guide RNAs. Type II Cas9 remains the most widely deployed dsDNA editor; Type V Cas12 nucleases provide crRNA‐only targeting, staggered dsDNA cleavage, and, in several cases, intrinsic crRNA‐array processing; and Type VI Cas13 systems act primarily on RNA [14, 15, 16, 27, 32]. For plant abiotic‐stress engineering, this classification matters because Type V systems combine practical editing features with delivery advantages: Cas12a is already a robust plant editor and transcriptional‐repression platform, while miniature Type V nucleases such as Cas12f and Cas12j/CasPhi reduce cargo size sufficiently to make viral, replicon‐based, and multiplex delivery more feasible [18, 19, 20, 21, 22]. Other CRISPR‐associated activities, including cyclic‐oligoadenylate signaling, CRISPR‐associated transposases, and foundation‐model‐discovered rare variants, are important for evolutionary understanding but are secondary to the argument of this Comment unless they directly improve plant delivery, multiplexing, or regulatory control [33, 34].

## 3 Classification and Evolutionary Relationships of the Type V CRISPR‐Cas System

Within Type V, Cas12 diversity can be organized by functional readiness for plant engineering rather than by exhaustive phylogeny (Figure 2). Canonical Cas12a and related medium‐sized nucleases are mature tools for plant editing, with well‐established TTTV‐like PAM recognition, crRNA‐array processing and multiplex editing or repression in several crops [14, 15, 16]. Miniature Cas12f/Cas14 proteins retain the RuvC nuclease core while reducing overall size to roughly 400–700 amino acids; early plant activity was modest, but guide and protein engineering, temperature tuning, and optimized expression have now produced practical mutagenesis in rice, tobacco, and other plant systems [18, 20, 44, 45, 46, 47, 48]. Hypercompact Cas12j/CasPhi proteins, first identified from huge phages, are also attractive because they are small, tracrRNA‐free, and compatible with compact delivery formats, and recent engineering has improved crop editing and base‐editing performance [19, 22, 41, 47]. By contrast, Cas12g is primarily an RNA‐targeting RNase; Cas12h functions as a crRNA‐guided nickase; Cas12lambda has been described as a self‐processing, compact dsDNA nuclease outside routine plant use; and TnpB‐like relatives represent promising but still early‐stage RNA‐guided nucleases [33, 36, 39, 49, 50, 51, 52]. This distinction is essential: not every compact nuclease discussed in the broader CRISPR literature has equivalent validation in plants. Consequently, the translational value of Type V diversity should be judged by plant‐active editing efficiency, delivery compatibility, guide simplicity, specificity, heritability, and the ability to support multiplex or regulatory editing in relevant crops.

## 4 Miniature CRISPR‐Cas12 Systems for Plant Genome Editing

Miniature Type V nucleases, including Cas12f, Cas12j/CasPhi, Cas12g, Cas12h, Cas12lambda, and TnpB‐like relatives, compress CRISPR function into smaller proteins than SpCas9 or many Cas12a orthologues, making them attractive for size‐limited plant vectors, viral replicons, and densely multiplexed guide designs. Their plant relevance, however, differs substantially across systems (Figure 2). Cas12a should be treated as the mature Type V benchmark rather than a miniature nuclease: it has been validated across many crops, processes its own crRNA arrays, and supports both editing and transcriptional repression [14, 15, 16]. Cas12f is the most advanced miniature DNA editor in plants. Cas12f variants have been tested in Nicotiana benthamiana, rice, tomato, and maize; initial activity was low, but protein engineering, improved guide scaffolds, and temperature optimization have increased indel formation and enabled demonstrations of stable, systemic delivery [18, 20, 44, 45, 46, 47, 48]. Cas12j/CasPhi has moved from low‐efficiency feasibility in plant replicons toward engineered systems with stronger genome editing and cytosine base editing in crops [19, 22, 41, 47]. Cas12g, in contrast, should not be presented as a heritable plant DNA editor because its known activity is RNA‐directed and is more relevant to transient RNA knockdown or antiviral concepts [50, 51]. Cas12h, Cas12lambda, and TnpB‐like nucleases should be described as emerging platforms whose biochemical properties are promising but whose deployment in plants is not yet equivalent to that of Cas12a, Cas12f, or engineered Cas12j [33, 36, 39, 49, 52]. This hierarchy clarifies the evidence base and prevents overstatement: compact Cas12 systems are already useful for plant delivery and rapid target testing, but only a subset currently supports stable, heritable crop editing with efficiencies suitable for trait development.

Recent studies further strengthen this protein‐engineering perspective. Engineered compact systems such as hpCasMINI, MiniCasUltra, and exoCasMINI improve Cas12f activity, specificity, PAM coverage, or editing efficiency, while Cas12j engineering has produced additional compact nuclease and base‐editing configurations [79, 80, 81, 82]. In plants, engineered TnpB variants now provide a related ultra‐compact benchmark, with activity reported in Nicotiana benthamiana, rice, and pepper, and viral delivery of an engineered TnpB enabling high‐efficiency, transgene‐free heritable editing [83, 84]. These studies should be interpreted as engineering advances that can inform the design of compact plant editing, while crop‐specific efficiency, heritability, and fidelity still require case‐by‐case validation (Table 1).

**TABLE 1:** Evidence status and translational readiness of compact and miniature Cas12‐related systems for plant genome engineering.

System	Approximate size / PAM / guide features	Plant validation status	Evidence base / activity	Delivery compatibility	Major limitation / appropriate interpretation
Cas12a/Cpf1	∼1,200‐1,400 aa; crRNA‐only; intrinsic crRNA‐array processing; TTTV‐like PAMs	Stable and transient editing validated in many plant species; also used for transcriptional repression	Robust benchmark for Type V plant editing and multiplex guide‐array processing [14, 15, 16]	Agrobacterium, protoplasts, and compact T‐DNA designs; less compatible with small RNA viral payloads than mini‐Cas	Mature Type V comparator rather than a miniature editor; larger cargo can restrict viral delivery
Cas12f/Cas14	∼400‐700 aa; compact RuvC nuclease; T‐rich PAMs; activity improved by scaffold and protein engineering	Validated in rice, tobacco/Nicotiana, tomato and maize through stable, protoplast or viral delivery assays	Plant activity has improved from low/modest to practical locus‐dependent editing; systemic PVX delivery shown for engineered AsCas12f [18, 20, 44, 45, 46, 47, 48, 54]	Highly attractive for PVX/TRV‐like vectors, geminiviral replicons and multiplex T‐DNA cassettes	Efficiency remains genotype‐, locus‐, temperature‐ and scaffold‐dependent; broad crop heritability needs continued validation
Cas12j/CasPhi	∼700‐800 aa; hypercompact phage‐derived nuclease; tracrRNA‐free; T‐rich PAMs	Transient plant delivery demonstrated; engineered variants now support crop editing and base editing	Initial replicon editing was low, but optimized Cas12j‐8/CasPhi systems show stronger plant performance [19, 22, 41, 47]	Fits compact viral or replicon payloads and can support multiplex prototyping	Promising but still less broadly benchmarked than Cas12a/Cas9; performance varies among variants and targets
Cas12g	∼700‐800 aa; RNA‐targeting RNase with collateral ssRNA activity	No established role as a heritable plant DNA editor	Relevant evidence supports RNA targeting and antiviral/knockdown concepts rather than genome editing [50, 51]	Potential transient RNA knockdown or antiviral applications	Should not be described as equivalent to DNA‐editing Cas12f/Cas12j systems
Cas12h	∼600‐900 aa; crRNA‐guided DNA nickase	Biochemical and non‐routine editing potential reported; plant validation remains limited	Nickase mechanism supports future paired‐nick, base‐editing or HDR‐priming concepts [36]	Could be useful for precision editing if plant‐active systems are developed	Emerging platform; claims should remain prospective until plant efficiency and heritability are shown
Cas12lambda	∼750 aa; compact dsDNA nuclease; self‐processing pre‐crRNA reported	Plant demonstrations are anticipated but not yet equivalent to Cas12a/Cas12f	Foundation‐model discovery expanded this family and simplified guide processing concepts [33, 52]	Potentially compatible with compact delivery and multiplex arrays	Currently a future plant‐engineering candidate rather than a validated crop‐editing tool
TnpB‐like / Cas12n‐related nucleases	Minimal RNA‐guided nuclease cores; often smaller than canonical Cas12 proteins	Early model‐system or biochemical activity; plant translation remains exploratory	Structural and evolutionary links explain the origin of compact Cas12 lineages [39, 49]	Very attractive for ultra‐small cargos if plant activity is optimized	Speculative for crop stress engineering until PAM, guide architecture, fidelity and plant expression are established

### 4.1 Delivery Methods and Advantages in Plants

One of the strongest advantages of miniature Cas12 systems is deliverability in plant cells compared with larger nucleases such as SpCas9 or many Cas12a orthologs. Delivery, rather than target discovery alone, is now a major bottleneck for translating multiplex genome engineering into elite germplasm. Many plant viral vectors and replicons impose strict cargo constraints, making large nuclease cassettes difficult to package or maintain, whereas compact editors such as Cas12f and Cas12j leave more space for promoters, guide arrays, regulatory domains, or repair templates [18, 19, 21]. Two plant demonstrations illustrate the significance of size. Gong et al. used deconstructed geminiviral replicons to deliver miniature Cas12j/CasPhi and Cas12f variants for transient virus‐induced editing in N. benthamiana, establishing delivery feasibility even though editing frequencies were low and not yet agronomically sufficient [19]. Ishibashi et al. then showed that a positive‐strand RNA virus vector can systemically deliver engineered AsCas12f and induce targeted mutagenesis in inoculated and distant tobacco tissues, highlighting how compact nucleases can unlock delivery routes inaccessible to larger editors [18]. Stable and protoplast‐based studies in rice, tomato, and maize further indicate that Cas12f activity can be improved by protein engineering, guide optimization, and heat treatment, but efficiency remains genotype‐, locus‐, and tissue‐dependent [44, 45, 46, 47, 48, 54]. Compact size also improves multiplex feasibility, as a mini‐Cas cassette plus several guide RNAs can be assembled into a single T‐DNA or viral payload more readily than with a Cas9‐based system. This feature is especially relevant for stress‐network engineering, where promoter edits, ORF edits, transcription‐factor perturbations, and transporter tuning may need to be tested in combinations. Nevertheless, delivery advantages should not be equated with field readiness. Viral and replicon systems can produce mosaic tissues, variable germline transmission, and complex segregation patterns, while high activity may increase the need for off‐target and structural‐variation screening. Therefore, the most realistic near‐term use of miniature Cas12 platforms is rapid target triage and network prototyping, followed by stable fixation of the best edit combinations through transformation, haploid‐inducer, or de novo meristem‐based pipelines [25, 26].

Non‐viral delivery should also be acknowledged. Recent plant nanodelivery studies describe carbon‐based, silica‐ or mesoporous, lipid/polymer, clay nanosheet, and metal‐organic framework carriers, as well as plant‐derived exosome‐like nanoparticles, as promising routes for delivering DNA, RNA, proteins, or CRISPR reagents across the plant cell wall [85, 86]. These approaches remain less mature than Agrobacterium, biolistics, or viral systems for heritable crop editing, but they are important because transient nanoparticle or extracellular‐vesicle‐like cargo delivery may reduce tissue‐culture dependence and support transgene‐free editing workflows.

## 5 Rewiring Multilayer Stress Networks in Plants With Compact Cas12 Systems

**FIGURE 3:**
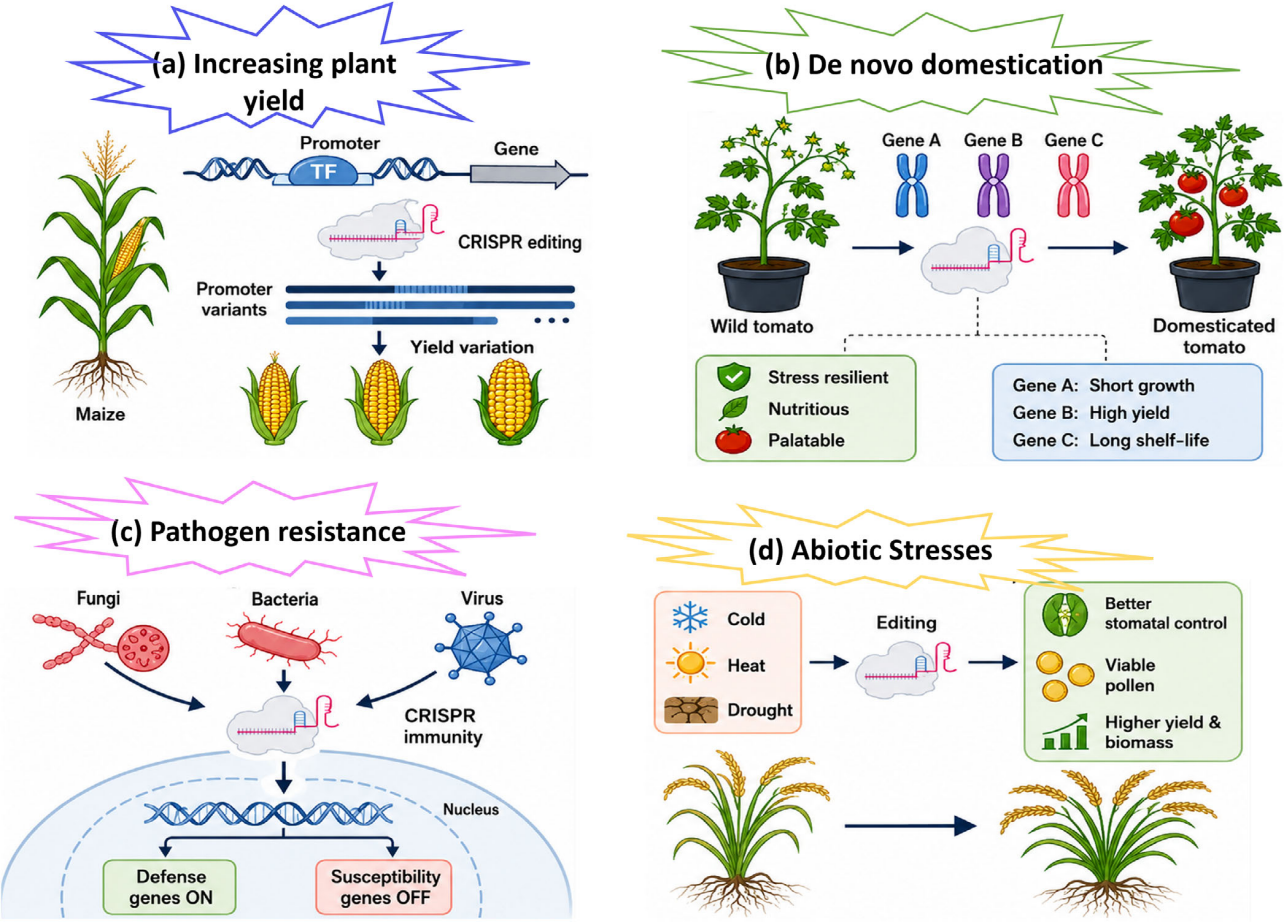
CRISPR‐based strategies for improving plant performance, domestication, and stress resilience. Schematic overview of major CRISPR‐enabled applications in crop improvement. (a) Promoter editing can generate allelic promoter variants that modulate developmental gene expression and create yield variation, illustrated here in maize. (b) Multiplex editing of domestication‐related loci can accelerate de novo domestication by converting wild tomato into improved lines with enhanced stress resilience, nutritional value, palatability, shorter growth cycle, higher yield, and longer shelf‐life. (c) CRISPR‐mediated pathogen‐resistance engineering can activate defense pathways by promoting resistance‐associated responses while suppressing susceptibility genes, thereby improving plant immunity against fungi, bacteria, and viruses. (d) Genome editing can also improve abiotic‐stress resilience by targeting stress‐response pathways associated with cold, heat, and drought tolerance, leading to better stomatal control, viable pollen, higher biomass, and improved yield. Overall, the figure highlights how CRISPR‐based editing links molecular regulation with agronomic trait improvement across yield, domestication, pathogen resistance, and abiotic‐stress adaptation. This image was created with BioRender (https://biorender.com/).

Plants survive abiotic stress through multilayer physiological networks that integrate ion homeostasis, ROS detoxification, osmotic adjustment, hormonal signaling, and transcriptional regulation. Compact Cas12 systems are valuable for this problem because they can, in principle, perturb multiple nodes in these networks within a single construct or delivery cycle. The central distinction is between evidence and design logic. Many stress‐tolerance examples in plants still rely on Cas9 or Cas12a, not on miniature Cas12 systems; these studies establish target classes and network principles, whereas compact Cas12 platforms may enable improved delivery or multiplex implementation. Accordingly, the following sections treat Cas9/Cas12a studies as validated biological evidence for stress‐network targets and miniature Cas12 systems as enabling technologies whose plant validation is strongest for Cas12f and engineered Cas12j, moderate for transient delivery assays, and still speculative for several newer compact nucleases. Figure 3 broadens the narrative from classification to trait design. The figure does not claim that every illustrated application has already been achieved with miniature Cas12. Instead, it shows what plant genome editing has already taught us about useful target classes. Promoter and cis‐regulatory editing can convert sequence variation into graded trait variation, as shown by drought‐yield improvement at ARGOS8 in maize and by systematic promoter editing in tomato [11, 76]. Multiplex editing can accelerate de novo domestication of wild or orphan crops by simultaneously changing plant architecture, flowering, fruit size, yield, and quality traits [53, 73, 74, 75]. Similar logic applies to pathogen resistance and abiotic‐stress adaptation: validated Cas9/Cas12a studies have identified susceptibility genes, antiviral targets, stomatal regulators [5, 9, 10, 50, 51, 57, 60, 53], ROS‐control nodes, and ABA‐linked drought‐response components that can serve as biological entry points for future compact‐Cas12 implementation.

### 5.1 Ion Homeostasis (Salt and Mineral Ion Balance)

**FIGURE 4:**
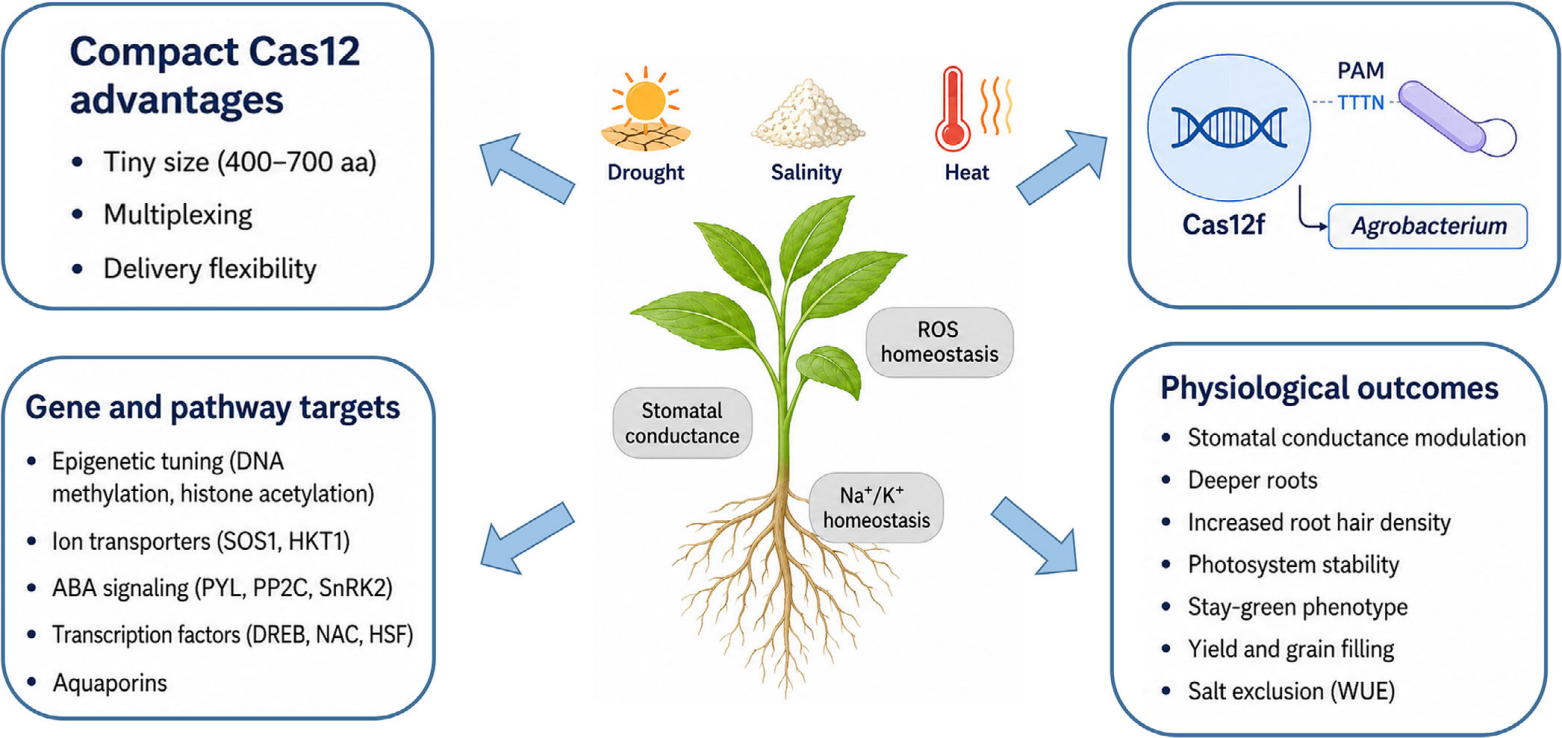
Compact Cas12‐enabled engineering of plant abiotic‐stress resilience. Conceptual model showing how compact Cas12 systems can support multilayer engineering of plant responses to drought, salinity, and heat stress. Compact Cas12 nucleases, exemplified by Cas12f, offer practical advantages including small protein size, multiplex editing capacity, and flexible delivery, including Agrobacterium‐mediated transformation. These features enable editing or regulation of key stress‐response targets, including epigenetic regulators, ion transporters, ABA‐signaling components, transcription factors, and aquaporins. The resulting network‐level changes can improve stomatal conductance, ROS homeostasis, and Na^+^/K^+^ balance, leading to deeper roots, enhanced root hair density, improved photosystem stability, stay‐green phenotype, better yield and grain filling, salt exclusion, and improved water‐use efficiency. This image was created with BioRender (https://biorender.com/).

Compact CRISPR‐Cas12 systems, including Cas12f and Cas12j, are able to target several ion homeostasis genes in a single construct. They can achieve more efficient and space‐saving multiplex editing than Cas9 or traditional Cas12a. Maintaining ionic balance under salt, drought, or heavy metal stress is critical. CRISPR‐mediated edits can enhance a plant's ability to regulate toxic ions (like Na^+^ or heavy metals) and retain essential ions (K^+^, Ca^+2^) as shown in Figure 4. For example, knocking out negative regulators of salt tolerance can boost ion transport capacity: in rice, *OsbHLH024* (a transcriptional repressor) was deleted using CRISPR, which up‐regulated key Na^+^/K^+^ transporters *OsHKT1;3*, *OsHAK7*, and the Na^+^ exporter *OsSOS1*, leading to improved salt tolerance [4]. Similarly, a CRISPR‐induced mutation in *OsRR22* (a type‐B response regulator in cytokinin signaling) increased rice salt tolerance without affecting growth [23]. Edits can also target ion channels and transporters directly: knocking out the rice cadmium transporter genes *OsNRAMP5* or *OsNRAMP1* via Cas9 reduced toxic Cd^2+^ accumulation in grains [55, 56]. Likewise, deletion of the rice R2R3 MYB transcription factor OsARM1 (a positive regulator of arsenic uptake) by CRISPR reduced arsenic accumulation. To create salt‐tolerant and heavy‐metal‐safe crops, Cas12a and related editors can multiplex targets in this ion homeostasis network – for instance, simultaneously editing a suite of transporters or regulators. A noteworthy strategy is promoter engineering in maize, where researchers replaced the native *ARGOS8* promoter with a stress‐inducible one using CRISPR, resulting in improved yield under drought conditions [11]. This concept could also be applied to ion transporter genes. Because Cas12a can process multiple guide RNAs from a single array, it is well‐suited to target multiple ion homeostasis genes at once, potentially stacking traits (e.g., enhanced Na^+^ efflux and K^+^ uptake). Editing ion transport networks using compact Cas12 systems has already been demonstrated in salt‐tolerant tomatoes, rice, and barley through gene knockouts, including *SlARF4* in tomato (auxin response factor) and *HvITPK1* in barley (inositol phosphate kinase), thereby improving salt tolerance by modulating ion and signaling balances [12]. Forward‐looking, compact Cas12 systems could be used to multiplex targets within this ion homeostasis network, potentially stacking traits such as enhanced Na+ efflux and K+ uptake.

### 5.2 ROS Detoxification and Antioxidant Defense

The small Cas12 systems provide the precise modulation of the ROS detoxification pathway through multiplexed editing and regulation of antioxidant genes, which is not possible with bigger systems such as Cas9 or typical Cas12a. Abiotic stresses often cause the accumulation of ROS, which damages cells. Plants mitigate oxidative stress via antioxidant enzymes (SOD, catalase, peroxidases) and stress‐response proteins. CRISPR editing can enhance this ROS‐scavenging network by removing suppressors of antioxidant activity or by upregulating protective genes. For instance, in tomato, CRISPR/Cas9‐mediated knockout of the stress‐responsive kinase SlMAPK3 enhanced heat tolerance by maintaining ROS homeostasis; the edited plants showed higher expression of antioxidant enzymes and heat‐shock proteins, thereby preventing excessive ROS buildup [5]. In rice, CRISPR edits of leaf morphology genes SRL1/SRL2 generated a “curled leaf” trait that not only reduced water loss but also enhanced ROS scavenging, improving drought tolerance [57]. Another example is the PQT3 gene in rice: loss‐of‐function mutants of Paraquat Tolerance 3 (generated by CRISPR) showed significantly improved growth under oxidative stress (paraquat treatment) and high salinity [58]. The PQT3 mutants indicate that removing a negative regulator can activate broad‐spectrum stress defenses, including antioxidant pathways. On the gene activation side, CRISPR‐based transcriptional activators have proven powerful. CRISPRa upregulation of AREB1 (a bZIP transcription factor controlling drought and oxidative stress genes) in Arabidopsis increased the plant's drought endurance by boosting its antioxidant enzymes and osmoprotectant sugars, thereby lowering ROS levels under water deficit [59]. In that study, the CRISPRa strategy used a dCas9‐histone acetyltransferase fusion, rather than dCas12, to enhance AREB1 expression and elevate the expression of downstream ROS‐defense genes [59]. Accordingly, the adaptation of compact Cas12f or Cas12j to equivalent CRISPRa or epigenetic activation formats should be presented as a prospective extension rather than as evidence already demonstrated by Roca Paixão et al. [59]. Compact Cas12f and Cas12j could be adapted similarly for epigenetic activation of antioxidant genes or CRISPR interference (CRISPRi) of ROS‐producing pathways, offering fine‐tuned control. Editing master ROS modulators using Cas12 systems (such as MAPKs, transcription factors, and enzymes) rewires the redox balance in plants, producing a more robust antioxidant defense that improves tolerance to heat, drought, salinity, and other ROS‐inducing stresses [5, 58]. Forward‐looking, compact Cas12f and Cas12j could be engineered to achieve epigenetic activation of antioxidant genes or CRISPR interference (CRISPRi) of ROS‐generating pathways, enabling precise control.

### 5.3 Osmotic Adjustment and Water Homeostasis

The small size of the Cas12 system allows more efficient multiplex gene editing of genes involved in osmotic adjustment and water homeostasis, enabling simultaneous changes in several pathways that would be difficult to achieve with Cas9 or typical Cas12a due to their size. Under drought or salinity stress, plants experience osmotic stress and cellular dehydration due to low water potential. To cope, they accumulate osmolytes (proline, sugars, betaines) and adopt morphological traits (e.g., rolled leaves, reduced stomatal density) to conserve water. CRISPR tools have enabled direct improvement of these osmotic adjustment mechanisms. A clear example is engineering stomatal traits: using CRISPR/Cas9, researchers edited the rice *OsDST* gene (which encodes a zinc‐finger transcription factor known to increase stomatal density and sensitivity) to produce mutant rice with lower stomatal density, wider leaves, and enhanced leaf water retention traits that significantly improved drought tolerance [10]. In parallel, the Semi‐Rolled Leaf genes (*SRL1* and *SRL2*) in rice were knocked out to induce a curled‐leaf phenotype that helps conserve moisture; these mutants had better water status and even showed increased ROS‐scavenging ability, linking morphology to osmotic and oxidative stress tolerance [57]. Another approach is to target genes involved in osmolyte synthesis or signaling. For instance, CRISPR knockout of *OsPYL9* (a redundant ABA receptor) in rice led to higher grain yield and drought tolerance [60], possibly by altering ABA sensitivity to avoid growth inhibition while maintaining osmotic adjustment. Likewise, mutating *OsERA1* (encoding a farnesyltransferase subunit that negatively regulates ABA responses) via CRISPR conferred drought resistance in rice [61]. *ERA1* mutants keep stomata more closed and accumulate osmoprotectants, reducing water loss. Promoter editing has also proven valuable: as mentioned, a promoter swap in maize (*ARGOS8* under a strong promoter) improved drought resilience in field conditions [11], likely by modulating ethylene signaling to reduce growth penalties during stress. This demonstrates that CRISPR can rewire how plants allocate resources under stress (maintaining yield with limited water). Future applications of compact Cas12 systems could include CRISPRa activation of osmolyte biosynthesis genes (for example, upregulating P5CS for proline production or TPS for trehalose) to increase osmotic adjustment capacity without requiring permanent DNA changes. Notably, CRISPR‐based domain edits allow precise control of protein function: in tomato, researchers deleted specific domains of the SlHyPRP1 cell‐wall protein (a proline‐rich protein) using multiplexed CRISPR/Cas9, creating alleles that improved tolerance to dehydration and salinity without growth defects [7]. This kind of fine‐tuning – removing a protein's regulatory domain showcases how gene editing can optimize osmotic stress responses. Editing genes that regulate water loss (stomatal regulators such as DST), water use (ABA signaling components), and osmolyte accumulation, compact Cas12a and its mini variants are poised to accelerate multi‐gene engineering to improve water homeostasis in crops. Forward‐looking, future uses of compact Cas12 systems may include Cas12a activation of osmolyte biosynthesis genes to enhance osmotic adjustment capacity without irreversible DNA alterations.

### 5.4 ABA and Hormonal Signaling Networks

The compact nature of Cas12 systems provides a distinct advantage for rewiring hormonal signaling networks, allowing the delivery of several guide RNAs in a single, compact construct, which is not easily achieved with bigger systems such as Cas9 or traditional Cas12a. Abscisic acid (ABA) and other phytohormones (such as ethylene, cytokinin, gibberellin, and auxin) orchestrate plant stress responses across the aforementioned layers. Rewiring hormonal signaling via CRISPR can yield plants that perceive and respond to stress more optimally. Many successful examples involve either enhancing ABA signaling (to close stomata and activate stress genes) or tempering growth‐inhibitory signals to preserve yield. On the ABA side, knockout of ABA negative regulators has been a common strategy. In rice, CRISPR/Cas9 editing of OsPYL9, which encodes an ABA receptor protein, improved drought tolerance and grain yield [60]. This result seems counterintuitive (since PYLs usually promote ABA responses). Still, it suggests that removing a single receptor can recalibrate the network to reduce ABA overreaction, thereby avoiding excessive growth suppression while maintaining drought resistance. Similarly, disrupting OsERA1 (a gene whose loss makes plants hypersensitive to ABA) made rice more drought‐hardy [61]. In wheat, simultaneous CRISPR editing of TaDREB2 and TaERF3 (two ABA‐inducible transcription factors) enhanced drought tolerance by activating downstream protective genes [13]. On the other hand, reducing growth suppression hormones can also help: the CRISPR‐Cas9 engineered ARGOS8 maize (with altered ethylene response) is one example, as it likely modulates cytokinin/ethylene crosstalk to keep plants growing under drought. In tomato, CRISPR was used to modify the gibberellin receptor gene SlGID1, yielding plants with higher leaf water content and improved drought resistance [62]. This suggests that tweaking GA signaling (which influences plant stature and stomatal development) can reduce water demand. Additionally, altering auxin and brassinosteroid pathways has shown benefits: knockout of SlARF4 (auxin response factor) in tomato boosted salt tolerance [12] and editing the brassinosteroid regulator BZR1 in tomato was reported to confer heat tolerance [63]. These hormone‐related targets often act as master switches that funnel environmental signals into appropriate physiological responses. Importantly, CRISPRa/CRISPRi tools enable modulation of hormone signaling without permanent gene knockout. For example, dCas9‐HAT‐mediated CRISPRa upregulation of AREB1 (ABA‐responsive element‐binding protein) can enhance the plant's intrinsic drought response [59]. Conversely, CRISPRi with dCas12 or dCas9‐KRAB repressors could transiently knock down genes like ABI1/ABI2 (ABA phosphatases) or specific PP2C clade members to enhance ABA sensitivity during stress, then release them afterward, creating a dynamic hormonal tuning. Although such CRISPRi strategies are still in the early stages, they hold promise for on‐demand stress tolerance. Targeting components of ABA signaling (receptors, kinases, phosphatases) and related hormone pathways (auxin/ERF/NAC TFs, ethylene modulators, etc.), genome editing can recalibrate the hormonal signaling layer. The result is plants that better balance growth and stress defense, for instance, maintaining yield under drought by judicious ABA responses achieved through both genetic mutations [13, 62] and transcriptional regulation [59]. Forward‐looking, compact Cas12‐based CRISPRa/CRISPRi tools can be used to dynamically tune hormone signaling for regulation without permanent gene deletion.

### 5.5 Transcriptional Regulation of Stress Responses

Compact Cas12 systems offer precision, multiplexed editing of transcription factors and regulatory RNAs, enabling coordinated changes in stress‐response networks more efficiently than Cas9 or standard Cas12a. At the top of the hierarchy, transcription factors (TFs) and regulatory RNAs coordinate multilayer stress responses by controlling gene expression. Our position is that this regulatory layer is the highest‐leverage entry point for climate‐resilience engineering because editing a single master switch can shift entire downstream programs at once, often more efficiently than stacking many structural‐gene edits. CRISPR enables precise modification of these regulators and, in principle, the introduction of beneficial natural alleles. Consistent with this “master‐switch” logic, CRISPR/Cas9 knockout of rice *OsNAC041* and *OsRAV2* produced plants with markedly improved salt tolerance [9, 64]. Likewise, editing *OsDST* (a repressor of stress responses despite its name) derepressed protective gene networks, yielding drought‐ and salt‐hardy plants, and disruption of the transcriptional repressor OsbHLH024 increased expression of multiple ion transporters, reinforcing how removal of a single suppressor can unlock coordinated stress‐defense outputs [4]. Post‐transcriptional regulators provide a parallel lever: deletion of *OsmiR535* lifted repression on its targets and contributed to enhanced salt tolerance, indicating that miRNA‐layer edits can reset expression equilibria toward adaptation [65].

Beyond irreversible knockouts, programmable regulation offers a way to tune network activity and potentially reduce fitness costs. dCas9‐HAT‐mediated CRISPRa upregulation of AREB1 in Arabidopsis activated ABA‐responsive programs and improved stress performance, illustrating how programmable activation can approximate a controlled “stress‐on” transcriptional state [59]. Conversely, CRISPRi‐based repression (e.g., dCas repressors) could enable graded knockdown of negative regulators rather than complete loss of function. Multiplex activation systems (e.g., CRISPR‐Act3.0) further highlight the direction of travel: delivering a single compact effector with multiple guides could coordinate induction of protective gene sets (LEA proteins, heat‐shock factors, antioxidant enzymes) on demand, and Cas12a's crRNA‐array processing is well‐suited to such multi‐gene control. Indeed, Cas12a has been used alongside Cas9 to edit stress‐related targets in parallel, generating enhanced tolerance in multiple crops [7, 66]. CRISPR editing and CRISPRa/i provide a coherent toolkit to rewire transcriptional networks that govern ion transport, redox balance, osmotic adjustment, and hormone signaling, with demonstrated gains in model and crop species [11, 64, 65]. Forward‐looking, heritable edits coupled to temporary epigenetic/transcriptional manipulation by compact Cas12 effectors can enable “stacked” resilience, stable removal of critical repressors, and conditional strengthening of defense circuits under extreme events.

## 6 Challenges, Trade‐Offs, and Deployment Roadmap

Compact Cas12 platforms expand the plant‐editing design space by relaxing delivery constraints and enabling dense multiplex perturbations that better match the polygenic architecture of abiotic‐stress tolerance. Their most compelling applications are not isolated knockouts, but combinations of regulatory editing, promoter rewiring, and programmable transcriptional or epigenetic control that create tunable stress buffers rather than constitutive defense switches. Three questions should guide translation. First, what does mini‐Cas12 enable that Cas9 or conventional Cas12a cannot? The clearest answer is compact delivery: smaller coding sequences improve compatibility with viral vectors, replicons, and multiplex constructs, and they may allow co‐delivery of effectors, guide arrays, and donor templates in formats that are difficult for larger nucleases [18, 19, 21]. Second, what evidence already supports this in plants? Cas12a is established for plant multiplex editing and transcriptional repression; Cas12f has demonstrated stable protoplast and viral delivery; and engineered Cas12j/CasPhi has advanced toward stronger crop editing and base editing [14, 15, 16, 18, 22, 44, 45, 46, 47, 48]. Third, what barriers must be solved before field translation? Efficiency remains variable across genotypes, loci, and tissues; systemic or transient delivery can generate mosaics; and multiplex stacks require rigorous fidelity assessment, including amplicon sequencing, targeted off‐target assays, and, for lead events, whole‐genome resequencing [72]. Growth‐defense trade‐offs also remain central. Abiotic‐stress engineering should therefore prioritize promoter or untranslated‐region edits, tissue‐specific expression, inducible CRISPRa/i, and buffered network tuning rather than constitutive activation of broad defense pathways. Finally, regulatory treatment of genome‐edited crops varies across jurisdictions, creating practical incentives to recover transgene‐free, well‐characterized small indels or promoter edits whenever possible [67, 68, 69, 70, 71]. The 2025–2026 regulatory landscape is changing rapidly: in the European Union, the NGT file is awaiting the European Parliament's second‐reading vote after Council progress on the first‐reading position; in China, 2025 reporting documents biosafety certificates for gene‐edited soybean, wheat, corn, and rice events, including the first gene‐edited rice event approved in December 2024; and in the United States, the 2020 SECURE rule was prospectively vacated in December 2024, APHIS reverted to legacy pathways during 2025, and USDA issued a May 2026 request for information to support possible future risk‐based rulemaking [67, 87, 88, 89]. A realistic deployment roadmap is therefore staged: use compact Cas12 delivery for rapid target triage; combine high‐throughput phenotyping with ion, ROS, osmolyte, and hormone readouts to rank edit combinations; fix the most promising alleles in elite backgrounds; and validate yield stability under controlled stress and field‐forward environments.

## 7 Conclusions

Compact Cas12 platforms, especially Cas12f and engineered Cas12j/CasPhi, provide a focused opportunity to accelerate plant abiotic‐stress engineering by linking compact delivery with multiplex perturbation of stress‐response networks. Their value lies in complementing, not replacing, Cas9 and Cas12a. Cas9 and Cas12a define many validated plant targets and remain robust editing platforms, whereas miniature Cas12 systems may make it easier to deliver, combine, and rapidly screen edits in ion transport, antioxidant control, osmotic adjustment, ABA signaling, and transcriptional regulation. The evidence base must be interpreted carefully: Cas12a and several Cas12f/Cas12j systems have meaningful plant validation, whereas Cas12g, Cas12h, Cas12lambda, and TnpB‐like nucleases remain promising but less mature for crop deployment. The next phase should move from catalog‐style descriptions of compact nucleases toward experimentally grounded design rules: which compact editor works in which tissue, at which temperature, with which guide architecture, and with what fidelity in elite germplasm. Recent advances in engineered compact nucleases, viral delivery, nanoparticle or extracellular‐vesicle‐like delivery concepts, and crop‐regulatory pathways sharpen this roadmap while emphasizing the need to align editor choice, delivery route, molecular characterization, and jurisdiction‐specific regulatory expectations from the design stage [67, 79, 80, 81, 82, 83, 84, 85, 86, 87, 88, 89, 77, 78]. If these barriers are overcome, compact Cas12 technologies can become powerful accelerators for stress‐network prototyping and for generating well‐characterized, transgene‐free crop alleles with improved resilience to salinity, drought, heat, and other climate‐linked constraints.

## Author Contributions

The manuscript was drafted by S.G.T., A.E., and A.M.A. and later revised by S.G.T., A.E., F.A.S., M.D., K.A., and A.M.A. Additionally, S.G.T. and A.M.A. originated the concept and were actively involved in interpreting the findings and supervising the work.

## Ethical Approval

This article does not contain any studies involving animals or human participants performed by any of the authors.

## Conflicts of Interest

The authors declare no conflicts of interest.

## Supporting information




**Supporting File**: ggn270040‐sup‐0001‐SuppMat.docx.
