# Supplementary material for: Compact Cas12 Systems for Multiplex Engineering of Plant Abiotic‐Stress Networks
Source: Adv Genet (Hoboken). 2026 Jun 15;7(2):e00067. doi: 10.1002/ggn2.202500067 (PMC13374822; doi:10.1002/ggn2.202500067)
Supplement: Supplementary file 1 — Supporting File: ggn270040‐sup‐0001‐SuppMat.docx. [file GGN2-7-e00067-s001.docx]

Record of Transparent Peer Review

Compact Cas12 Systems for Multiplex Engineering of Plant Abiotic-Stress Networks

Data submitted: 21-Nov-2025

Accepted: 04-June-2026

**Response to Reviewer #1- Round 1**

We thank the reviewer for the constructive and detailed feedback. Below, we respond point-by-point. All changes in the revised manuscript are highlighted in red and tracked.

**Comment 1:** The manuscript predominantly summarizes previous studies without providing critical evaluation or novel insight expected for a commentary; limitations and unresolved questions are not sufficiently discussed.

**Response:** We reframed the article explicitly as a perspective/commentary and added critical analysis that identifies the key bottlenecks limiting translation of mini-Cas12 editors to crops (efficiency–fidelity trade-offs, temperature and genotype dependence, delivery-induced mosaicism, and regulatory/off-target expectations). We also added a field-translation roadmap that converts these bottlenecks into actionable design rules and validation steps.

*Changes in manuscript:* Abstract rewritten to articulate the commentary thesis and to foreground bottlenecks and a practical roadmap. A new section, 'Challenges, trade-offs, and deployment roadmap,' was added immediately before the Conclusion, with targeted citations (e.g., Wolt et al. 2016; Entine et al. 2021; Voytas and Gao 2014; Gao 2021).

**Comment 2:** The manuscript reiterates published findings without synthesizing a novel conceptual framework or highlighting specific gaps.

**Response:** We agreed and addressed this by proposing a unifying framework — 'stress-network prototyping' — that prioritizes regulatory hotspots, multiplex perturbations of network hubs, and an iterative triage-to-fixation workflow (viral delivery for rapid screening → stable fixation of elite allele combinations → transgene-free segregation). This framing clarifies what compact Cas12 uniquely enables relative to Cas9/Cas12a and where the remaining gaps are.

*Changes in manuscript:* Added a 'Perspective and scope' paragraph at the end of the Introduction that states the position and the design rules focus. Added a dedicated roadmap section that outlines staged validation and translation steps (Ellison et al. 2020; Maher et al. 2020; Ishibashi et al. 2024).

**Comment 3:** The text is dense with technical details, gene names, and pathways, reducing clarity and obscuring the main argument.

**Response:** We streamlined narrative passages by consolidating repeated gene lists into functional modules and emphasizing principles (what to edit, how to multiplex, and how to validate) rather than enumerations. Where gene examples remain, they are used illustratively to support a design principle.

*Changes in manuscript:* Abstract condensed and restructured. Added an explicit scope statement in the Introduction. Minor edits throughout to consolidate gene lists into modules and to improve readability; key new synthesis is captured in the roadmap section.

**Comment 4:** Sections are long and read like a review; the manuscript lacks a concise thesis/position.

**Response:** We strengthened the statement and adjusted the structure so that descriptive background supports a clear argumentative arc: (i) what mini-Cas12 uniquely enables, (ii) what remains technically limiting, and (iii) an actionable workflow for research and translation.

*Changes in manuscript:* Added thesis/scope paragraph at the end of the Introduction. Added a new 'Challenges, trade-offs, and deployment roadmap' section before the Conclusion; Conclusion revised to reflect a balanced perspective and translation conditions.

**Comment 5:** Ethical, regulatory, and technical challenges (off-targets, field constraints, delivery limitations) are not addressed in depth.

**Response:** We expanded the discussion of fidelity and off-target risk management, mosaicism from transient/viral delivery, and the heterogeneity of regulatory pathways across jurisdictions that affects optimal engineering choices.

*Changes in manuscript:* New roadmap section includes subsections on fidelity/off-target management (Wolt et al. 2016), delivery/mosaicism and staged translation (Ellison et al. 2020; Maher et al. 2020), and regulatory landscape (Entine et al. 2021; Voytas and Gao 2014).

**Comment 6:** Some sentences are overly long and complex; grammatical corrections are needed; the abstract mirrors the introduction rather than providing a perspective.

**Response:** We edited the Abstract to provide a concise perspective and improved sentence-level clarity by shortening overly long sentences and reducing repetition where feasible.

*Changes in manuscript:* Abstract replaced with a clearer, perspective-driven version. Targeted sentence-level edits were applied throughout the Introduction and concluding sections; all edits are marked in red.

**Reviewer #2**

The article summarizes information about Cas enzyme classification, their evolutionary history, effectiveness, and mini cas12 applications in developing stress-tolerant crops. But this article may not be appropriate to classify as a "Commentary" as it does not offer critical perspectives, expert insights, or discussion of a specific recently published study. Instead, it reads more like a comprehensive review that compiles and summarizes previous findings without providing new interpretations or meaningful commentary.

**Response to Reviewers- Round 2**

Dear Editor and Reviewers,

We sincerely thank the reviewers for their constructive and careful evaluation of the revised manuscript. We have substantially revised the manuscript, with all newly added or corrected text highlighted in red in the revised file. The revision now further condenses the taxonomic background, explicitly separates established plant evidence from prospective compact-Cas12 applications, integrates the revised figures into the manuscript text, and adds detailed figure legends with recent and direct references.

The figures were redrawn and corrected in response to the reviewer comments using BioRender and PowerPoint, and the manuscript now cites each figure at the appropriate location rather than treating them as separate material.

**Reviewer #1**

**Comment:** Sections 2 and 3 remain disproportionately long for a perspective/commentary and should be condensed to a single paragraph or compact table without loss of argumentative value.

**Response:** We agreed that an exhaustive CRISPR-Cas taxonomy is not the central contribution of this Comment. We therefore kept Sections 2 and 3 concise and explicitly tied them to the manuscript thesis: why compact Type V/Cas12 effectors matter for plant abiotic-stress engineering. Rather than providing a broad review of CRISPR evolution, the revised text now uses the updated classification only to orient readers to Class 2 Type V systems and to distinguish mature plant platforms from emerging compact systems.

*Revision made:* Sections 2 and 3 have been condensed and refocused. Section 2 now summarizes the two-class/seven-type CRISPR-Cas framework using the updated Makarova et al. classification [27], and Section 3 now organizes Type V/Cas12 systems by plant-engineering readiness rather than exhaustive phylogeny. The revised Figures 1 and 2 are cited directly in these sections and supported by recent references [14–22,27,35–49,68].

Table 1 has been revised to include compact-editor size, PAM/guide features, plant validation status, delivery compatibility, and activity interpretation. Where precise editing efficiencies differ strongly by species, locus, temperature and guide scaffold, the table reports representative activity qualitatively and directs readers to the cited primary studies.

**Comment:** The scope paragraph uses informal phrasing and should be removed or substantially rewritten.

*Revision made:* The Introduction now states the central thesis directly: compact Cas12 systems complement Cas9/Cas12a by enabling delivery-efficient, multiplex perturbation of abiotic-stress networks in elite germplasm. The redundant scope paragraph has been eliminated, and all added text is highlighted in red.

**Reviewer #5**

**Major comments**

**Comment:** All figures need to be redrawn carefully and precisely, cited correspondingly in the manuscript, and corrected for unclear classification, typing errors, duplicate labels and inconsistent colors. Figure 5 is visually busy and should be replaced by sharper conceptual figures.

**Response:** We fully agreed and comprehensively revised the complete figure set. All figures were redrawn in a cleaner and more consistent style, with corrected labels, standardized color coding, clearer typography, and detailed captions. We also added or strengthened in-text figure citations so that each figure is integrated into the manuscript argument rather than separated from the text.

*Revision made:*

- Figure 1 was redrawn as an updated two-class/seven-type CRISPR-Cas classification schematic, following the recent classification by Makarova et al. [27]. It now clearly separates Class 1 systems (Types I, III, IV and VII) from Class 2 systems (Types II, V and VI) and lists representative signature effectors and targets.
- Figure 2 was redrawn as a simplified CRISPR-Cas classification framework with representative gene architecture, signature effectors, target molecules and functional notes. The Type V/Cas12 portion now avoids implying equivalent validation among Cas12a, Cas12f, Cas12j/CasPhi, Cas12g, Cas12h, Cas12lambda and TnpB-like systems.
- Figure 3 was simplified into four high-contrast panels summarizing promoter editing for yield, de novo domestication, pathogen resistance and abiotic-stress improvement. The figure is now used as contextual evidence for CRISPR-enabled target classes, not as direct evidence that all applications have been achieved with miniature Cas12.
- Figure 4 was replaced with a sharper compact-Cas12 conceptual model focused on abiotic-stress resilience. The incorrect Cas12f spelling was corrected, duplicate labels were removed, color coding was standardized, and the figure now links compact Cas12 advantages, target classes and physiological outcomes.

**Comment:** The manuscript is too clumsy and review-like. It should be substantially shortened and refocused around three questions: (1) what does mini-Cas12 uniquely enable in plants that Cas9/Cas12a do not, (2) what evidence supports this in plant systems, and (3) what technical barriers remain before field translation?

**Response:** We agreed that a Comment article should present a focused argument rather than a catalog of background information. The revision now explicitly uses these three questions to structure the argument and the deployment roadmap.

*Revision made:* The general classification sections were shortened, the roadmap section was sharpened, and the manuscript now states the three questions directly in Section 6. The revised text emphasizes: (i) compact delivery and multiplex compatibility as the main distinctive value of mini-Cas12; (ii) current plant evidence for Cas12a, Cas12f, and engineered Cas12j/CasPhi; and (iii) unresolved barriers including efficiency, fidelity, mosaicism, off-target/structural variation, genotype dependence, and regulatory requirements.

**Comment:** Most plant applications cited are established with CRISPR-Cas9 rather than miniature Cas12, and the manuscript should explicitly separate systems demonstrated in stable plants, transient assays/protoplasts, outside plants or speculative future directions. A concise table should summarize nuclease size, PAM, guide architecture, plant validation status, evidence base, delivery compatibility, editing efficiency range and limitations.

**Response:** We agreed. This was one of the most important revisions. We now explicitly distinguish validated evidence for biological targets from evidence for the compact-Cas12 platform. We also avoid presenting Cas9/Cas12a examples as if they were miniature Cas12 demonstrations.

*Revision made:* Table 1 has been expanded and revised to include approximate size, PAM/guide features, plant validation status, established evidence-based/representative activity, delivery compatibility, and major limitations. The table separates: Cas12a as a mature Type V plant benchmark; Cas12f as the most advanced miniature DNA editor in plants; engineered Cas12j/CasPhi as a promising hypercompact plant editor; Cas12g as an RNA-targeting platform; and Cas12h, Cas12lambda, and TnpB-like nucleases as emerging or prospective tools requiring further plant validation. In the main text and figure legends, Cas9/Cas12a examples are now explicitly described as target-class evidence rather than direct miniature-Cas12 evidence.

**Minor comments**

**Comment:** Direct primary citations should be used instead of secondary citations, including the statement that salinization affects approximately 20% of irrigated land.

**Response:** We agreed and replaced secondary citation support with a direct reference. We also checked the manuscript carefully for central claims and citation alignment.

*Revision made:* The salinization statement is now supported by Singh [1]. We also strengthened figure legends and key claims with direct references, including the updated CRISPR-Cas classification [27], Cas12f/Cas12j plant studies [18–22,44–48], and de novo domestication studies [75–77].

**Comment:** Full names of abbreviations should be provided at first mention, for example SAPs.

**Response:** We agreed and reviewed the manuscript, spelling out abbreviations at first mention where needed.

*Revision made:* The revised text now introduces reactive oxygen species (ROS), stress-associated proteins (SAPs), abscisic acid (ABA), transcription factors (TFs), protospacer-adjacent motif (PAM), homology-directed repair (HDR), non-homologous end joining (NHEJ) and water-use efficiency (WUE) in a clearer manner.

**Comment:** There are citation mistakes, including the use of a dCas9/TALE activation citation to support dCas12 claims and incorrect citation of the Gong et al. plant mini-Cas delivery study. Every central citation should be checked.

**Response:** We agreed and corrected the affected statements. We no longer use the plant CRISPR-Act citation as evidence for dCas12a activation. Instead, the text distinguishes Cas12a transcriptional repression in plants from CRISPR-Act activation platforms. The Gong et al. and Ishibashi et al. citations are now used specifically and accurately for miniature Cas12j/CasPhi/Cas12f geminiviral delivery and systemic engineered AsCas12f delivery, respectively.

*Revision made:* Citation-supported claims in the Introduction, Sections 3–4, and Table 1 were revised. The manuscript now states that Cas12a supports plant editing and transcriptional repression [14–16], while CRISPR-Act systems support plant activation more generally [17]. Evidence for compact Cas12 delivery is now attributed to Gong et al. [19] and Ishibashi et al. [18].

**Comment:** Use one spelling consistently, for example hypercompact rather than alternating with hyper compact.

**Response:** We agreed and standardized spelling throughout.

*Revision made:* The term "hypercompact" is used consistently throughout the manuscript, response letter and figure captions.

**Comment:** The title is catchy but slightly vague. Consider a more precise title such as "Compact Cas12 Systems for Multiplex Engineering of Plant Abiotic-Stress Networks."

**Response:** We agreed with the reviewer and adopted the suggested direction.

*Revision made:* The title has been changed to: "Compact Cas12 Systems for Multiplex Engineering of Plant Abiotic-Stress Networks."

Overall, we appreciate the reviewers' guidance, which substantially improved the manuscript. The revised manuscript now presents a more focused, evidence-aware, and figure-integrated Comment article. All newly added or corrected manuscript text is highlighted in red, and the updated figure set and figure captions are aligned with the revised text and reference list.

**Response to Reviewers- Round 3**

**Editor's Comment 1:** As noted in our previous decision regarding editor's comments, the individual subsections continue to be organized primarily around what CRISPR editing broadly has achieved at each stress layer, with compact Cas12 applications appearing as brief additions at the end of each subsection. We do not require a full rewrite of these subsections. Rather, we want the authors to add a focused opening sentence to each of Sections 5.1–5.5 that explicitly articulates what compact Cas12 specifically enables at that stress layer that Cas9 or conventional Cas12a cannot. Additionally, any prospective compact Cas12 applications currently positioned at the end of each subsection should be moved to a more prominent location within the subsection, clearly labeled as forward-looking.

**Response:** We added a focused opening sentence to each section from 5.1 to 5.5. We have also written "forward-looking" prospective compact Cas12 applications at the end of each and moved other statements to a more prominent location within that subsection. The new sentences are highlighted in yellow in the revised manuscript.

**Editor's Comment 2:** Our previous decision letter specifically requested that the reference list be updated to capture 2025–2026 advances, for instance:

- Protein-engineered mini-Cas12 variants reported in 2025–2026
- Nanoparticle and extracellular vesicle delivery approaches for plant genome editing
- Recent regulatory developments governing genome-edited crops in the EU (including the NGT regulation legislative progress), China, and the United States (SECURE rule updates)

**Response:** We expanded the manuscript and the reference list to capture these advances from 2025–2026. Specifically, we added recent engineered compact-nuclease references, including hpCasMINI, MiniCasUltra, exoCasMINI, engineered Cas12j, and engineered/virally delivered TnpB systems [81–86]. We also added text and references on nanoparticle and extracellular-vesicle-like delivery concepts for plant genome editing [87,88]. Finally, the regulatory section has been updated to reflect current EU NGT legislative progress, China's recent genome-edited crop approvals/biosafety certificates, and the United States post-SECURE transition following the 2020 rule vacatur and the May 2026 USDA request for information [69,89–91]. All newly inserted or revised text and new references are highlighted in red in the revised manuscript.

**Editor's Comment 3:** Our previous decision letter stated that AI-generated figures are not acceptable and that figures must be directly produced or supervised by the authors. The revision response letter notes that figures were redrawn using BioRender, which can be updated in the figure legend.

**Response:** In the revised version, all figures generated with BioRender include the following text: "This image was created with BioRender (<https://biorender.com/>)."

**Editor's Comment 4:** The following smaller issues should also be addressed in the revision:

- Reference [69] (Chen et al., Science 2018) is cited in the context of regulatory and fidelity considerations but describes Cas12a collateral DNase activity, which is not directly relevant to that context.
- Roca Paixão et al. [59] used dCas9.
- The Data Availability Statement can be removed since there is no new data.

**Response:** We agreed and corrected both points. The description of Roca Paixão et al. [59] now states that the study used a dCas9-histone acetyltransferase fusion, not dCas12. We also revised the related statements in the ABA/hormone-signaling and transcriptional-regulation sections so that [59] is not presented as evidence for dCas12-based activation. In addition, Chen et al. (Science 2018) has been removed from the regulatory/fidelity context. Reference [69] has been replaced with a current EU NGT legislative source, and the fidelity sentence now cites the off-target/fidelity source [74] rather than the unrelated collateral-DNase paper. All corrected text and the updated reference [69] are highlighted in red in the revised manuscript.

We removed the Data Availability Statement in the revised manuscript.

**Response to Reviewers- Round 4**

**Editor’s Comment:** One small thing worth checking before acceptance: the abstract still reads "This review paper offers a forward-looking perspective..." — given the article type is listed as "Comment," please align it with the terminology, for instance in the abstract: We offer a forward-looking roadmap in which compact Cas12 systems complement Cas9 and Cas12a by enabling rapid target triage, promoter and untranslated-region engineering, transient CRISPR activation/interference, and stepwise assembly of durable sentinel edits.

**Response**: Thanks for the comment. We have added the new sentence based on your suggestion. The new sentences are highlighted in yellow in the revised manuscript.
